# Interplay Between NF‐κB and Kruppel‐like Factors in Vascular Inflammation and Atherosclerosis: Location, Location, Location

**DOI:** 10.1161/JAHA.113.000290

**Published:** 2013-06-21

**Authors:** G. Brandon Atkins, Daniel I. Simon

**Affiliations:** 1 Harrington Heart & Vascular Institute, Case Cardiovascular Research Institute, Department of Medicine, University Hospitals Case Medical Center, Case Western Reserve University School of Medicine, Cleveland, OH (B.A., D.I.S.)

**Keywords:** editorials, atherosclerosis, KLF, NF‐κB, vascular inflammation

## Introduction

Atherosclerosis is a highly complex process involving the dynamic interplay between cell types intrinsic (eg, endothelial cells [ECs] and smooth muscle cells [SMCs]) and extrinsic (eg, lymphocytes and myeloid cells) to the blood vessel wall.^1^ Over the last several decades extensive work has been conducted that details the many molecular pathways involved in mediating the pathologic vascular inflammation that occurs in atherosclerosis. One of the most important factors in the regulation of this process is the transcription factor nuclear factor κB (NF‐κB). NF‐κB has been shown to regulate numerous genes involved in the initiation and progression of atherosclerosis, including genes involved in inflammation and immune cell regulation as well as apoptosis and cell proliferation.^2–3^ Genetic approaches have been utilized to identify the different mechanisms and cell type intrinsic actions of NF‐κB in mediating atherosclerosis and vascular inflammation. In providing a new example, Yoshida and colleagues^4^ in this issue of *Journal of the American Heart Association* selectively inhibit the NF‐κB pathway in SMCs and report attenuation of SMC phenotypic switching and neointima formation after vascular injury. The potential importance and impact of this study requires an understanding of our current knowledge of NF‐κB action in other vascular cells.

In an effort to assess in vivo the EC‐specific function of NF‐κB signaling in the pathogenesis of atherosclerosis, Gareus and colleagues generated several different mouse models employing either EC‐specific ablation of NEMO/IKKγ to interfere with IKK activation or transgenic expression of degradation resistant dominant negative IκBα superrepressor (DNIκBα). EC‐restricted inhibition of NF‐κB, in Apolipoprotein E knockout (ApoE^−/−^) mice fed a high fat western diet resulted in greatly reduced size and severity of atherosclerotic lesions. Inhibition of NF‐κB abrogated expression of adhesion molecules, diminished expression of cytokines and chemokines, and impaired macrophage recruitment to atherosclerotic plaques.^5^ These findings support a critical role of the endothelial NF‐κB pathway in promoting the pathogenesis of atherosclerosis. Findings from the de Winther laboratory using macrophage‐specific inhibition of the NF‐κB pathway suggest that the role of NF‐κB in this cell type is not as straightforward. Surprisingly, initial studies using LDL receptor deficient (LDLR^−/−^) mice with a macrophage‐specific deletion of IκB kinase 2 (IKK2), showed increased atherosclerosis with more advanced lesions and more necrotic plaques.^6^ These results highlight the importance of NF‐κB in the gene program promoting the resolution of inflammation. In contrast, a subsequent study using bone marrow from p50 deficient mice transplanted into irradiated LDLR^−/−^ mice demonstrated that hematopoetic deficiency of the p50 subunit of NF‐κB results in smaller atherosclerotic lesions with a near complete absence of foam cells.^7^ A more recent study in LDLR^−/−^ mice transplanted with bone marrow from mice with myeloid‐specific deletion of IκBα demonstrated larger and more advanced atherosclerotic lesions through enhanced leukocyte recruitment to plaques.^8^ These disparate results of NF‐κB function in macrophages have yet to be fully reconciled. Despite the in vivo study of the role of the NF‐κB pathway in atherosclerosis and vascular inflammation in ECs and macrophages, similar studies investigating this pathway in vascular SMCs have been lacking to date.

In this issue of *Journal of the American Heart Association*, Yoshida and colleagues^4^ investigate the cell autonomous role of NF‐κB within SMCs by using SM22α Cre to generate mice with SMC‐specific expression of a truncated form of IκB (IκBΔN) lacking 2 N‐terminal phosphorylation sites. SMC‐specific expression of IκBΔN results in selective sequestration of NF‐κB in the cytoplasm of SMCs. Mice with SMC‐specific inhibition of the NF‐κB pathway displayed a significant reduction in neointima, slower proliferation rate, and an attenuated inflammatory response after vascular injury as demonstrated by decreased expression of *VCAM‐1*,* ICAM‐1*, and *CCL20*. Down‐regulation of SMC differentiation markers and myocardin is a hallmark feature of the phenotype switching of differentiated SMCs in response to vascular injury. SMC‐selective inhibition of NF‐κB resulted in attenuated repression of SMC differentiation factors, SM α‐actin, SM22α, and SM myosin heavy chain (SMMHC) as well as myocardin. Mechanistically, the authors demonstrate that IL‐1β‐mediated decrease in expression of SMC differentiation markers and myocardin is dependent on NF‐κB and is partially mediated by Kruppel‐like Factor 4 (KLF4). Finally, detailed myocardin promoter analysis reveals consensus binding sites for NF‐κB and KLF4 and chromatin immunoprecipitation (ChIP) analysis in cultured SMCs and injured vessels demonstrates that both p65 and KLF4 are bound to the myocardin promoter in response to an inflammatory cytokine or vascular injury. These novel findings clearly identify the NF‐κB pathway in SMCs as being critical for mediating the SMC response to vascular inflammation.

Interestingly, in SMCs, NF‐κB acts as a direct transcriptional repressor of the myocardin promoter in response to vascular injury and inflammation. During vascular inflammation and atherosclerosis in other cell types, such as ECs and macrophages, NF‐κB acts predominantly as a direct transcriptional activator via interaction with major coactivator proteins such as CREB‐binding protein (CBP), p300, steroid receptor‐coactivator‐1 (SRC‐1), and p300/CBP‐associated factor (PCAF).^2–3,9^ This apparent unique tissue‐specific transcriptional repression by NF‐κB in SMCs in response to vascular injury warrants further investigation to elucidate the detailed molecular mechanisms.

Also very intriguing is the identification of KLF4 as a cooperative transcriptional inhibitory binding partner with NF‐κB on the myocardin promoter in SMCs, resulting in the down‐regulation of SMC differentiation markers. KLFs are a subgroup of the zinc finger family of transcription factors that have been shown to broadly regulate numerous physiological and pathological processes in many cell types and organ systems.^10^ Remarkably, in the past decade KLFs have been identified as atheroprotective factors in multiple cell types including ECs, macrophages, and SMCs.^11^ KLF2 and KLF4 are highly expressed in ECs and both have been implicated as “molecular switches” regulating endothelial health and disease by differentially controlling the expression of factors that confer antiinflammatory, antithrombotic, vasodilatory, and antiproliferative effects in ECs.^12^ One important mechanism of action of KLF2 and KLF4 in ECs is the inhibition of cytokine‐mediated induction of cell adhesion molecules. In striking contrast to the observations of Yoshida and colleagues with KLF4 in SMCs, KLF2 and KLF4 in ECs inhibit NF‐κB function by competing for the common coactivators p300 and PCAF,^13–14^ thereby serving as inhibitors of vascular inflammation. Notably, this function is independent of KLF DNA binding. More recently KLF11 has been shown to be a suppressor of endothelial inflammatory activation by a very similar NF‐κB inhibitory mechanism.^15^

KLF2 and KLF4 are also highly expressed in monocytes/macrophages where they have been shown to play important roles in monocyte differentiation, macrophage activation and polarization, and regulation of inflammatory signaling.^11^ As in ECs, a major mechanism of action of KLF2 and KLF4 in monocytes/macrophages is the inhibition of the NF‐κB pathway.^16–17^ Importantly, in vivo studies from our group and others using the ApoE^−/−^ and LDLR^−/−^ models have established KLF2 and KLF4 as atheroprotective factors in both endothelial and myeloid cells.^18–21^ Taken together these findings suggest that interplay between NF‐κB and KLFs is cell‐type specific and is important for mediating the cellular response to vascular inflammation (see Figure). Future studies aimed at investigating the cell‐specific mechanism that determines whether the interaction between KLFs and NF‐κB is cooperative or inhibitory would be of great interest in the field.

**Figure 1.**
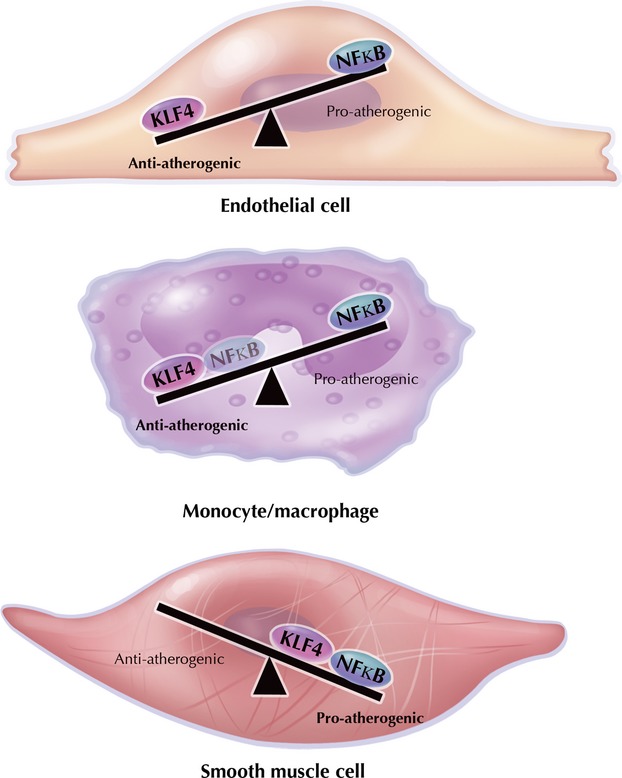
Interplay between NF‐κB and KLFs in atherosclerosis and vascular inflammation is cell type specific. Schematic diagram demonstrating antagonism between KLF4 and NF‐κB in endothelial cells and monocytes/macrophages vs cooperation in smooth muscle cells. NF indicates nuclear factor; KLFs, Kruppel‐like factors.

KLFs are also expressed and play key roles in SMC biology. Work from the Owens lab has outlined the importance of KLF4 in mediating the SMC response to vascular inflammation.^11^ Yoshida and colleagues highlight the necessity for cooperation between KLF4 and NF‐κB in mediating the inhibition of myocardin in response to vascular injury.^4^ The authors choose to focus on KLF4 for their studies as KLF4 is induced in SMCs by IL‐1β. Notably, KLF5 and KLF15 are 2 other KLFs that have been demonstrated to play key roles in mediating the SMC response to vascular inflammation^11,22^ and warrant investigation in this model. Finally, in order to truly assess the cell‐autonomous role of NF‐κB in SMCs in the pathogenesis of atherosclerosis, it will be important to cross mice with SMC‐specific inhibition of the NF‐κB pathway to the LDLR^−/−^ or ApoE^−/−^ mouse models of atherosclerosis. Along the same lines, similar in vivo studies with SMC‐specific deletion of KLF4, KLF5, and KLF15 in atherosclerosis models have yet to be conducted. Such studies would be highly revealing and provide novel mechanistic insight into the pathogenesis of atherosclerosis, thereby potentially resulting in novel therapies for disease.
